# Size and Zinc Substitution Govern the MPI and MRI Performance of Ferrite Nanoparticle Tracers

**DOI:** 10.1021/acsmaterialsau.6c00147

**Published:** 2026-10-24

**Authors:** Bahareh Rezaei, Hanlei Wang, Marielena Molinares Ponce, Md Shahriar, Karla Mercedes Paz González, Linh Nguyen T. Tran, Vinit Kumar Chugh, Changxue Xu, Jenifer Gómez-Pastora, Rui He, Kai Wu

**Affiliations:** † Department of Electrical and Computer Engineering, 6177 Texas Tech University, Lubbock, Texas 79409, United States; ‡ Department of Physics, 7831 University of South Florida, Tampa, Florida 33620, United States; § Department of Industrial, Manufacturing, and Systems Engineering, Texas Tech University, Lubbock, Texas 79409, United States; ∥ Department of Chemical Engineering, Texas Tech University, Lubbock, Texas 79409, United States; ⊥ 116171 Seagate Technology LLC, Shakopee, Minnesota 55379, United States; # Department of Physics, 12329 The University of Texas at Arlington, Arlington, Texas 76019, United States

**Keywords:** Magnetic nanoparticles, magnetic particle imaging, magnetic resonance imaging, zinc ferrite, magnetic particle spectroscopy, T1 contrast agents

## Abstract

Magnetic nanoparticles (MNPs) that can support both magnetic particle imaging (MPI) and magnetic resonance imaging (MRI) are attractive for dual-modal imaging because they combine the quantitative, background-free tracer detection of MPI with the anatomical contrast of MRI. However, the MPI- and MRI-relevant performance of ferrite nanoparticles is strongly governed by core size, composition, magnetic relaxation, and biological environment. Here, we report a comparative study of four lab-synthesized, quasi-cubic MNP formulations: 30 nm Fe_3_O_4_ nanoparticles (F1), 10 nm Fe_3_O_4_ nanoparticles (F2), 30 nm Zn_0.4_Fe_2.6_O_4_ nanoparticles (Z1), and 10 nm Zn_0.4_Fe_2.6_O_4_ nanoparticles (Z2). The morphological, physicochemical, cytocompatibility, and cellular uptake properties of these nanoparticles were systematically characterized. Magnetic particle spectroscopy (MPS) was used as an MPI-relevant screening method to compare the dynamic magnetization responses of these nanoparticles under excitation fields in both water and agar phantoms. Z1 produced the strongest mass-normalized harmonic amplitudes under 7.75 kHz excitation in both water and agar phantoms, identifying it as the highest signal formulation for MPI applications. Agar embedding generally reduced the harmonic amplitudes, consistent with restriction of Brownian rotation and demonstrating that physical confinement can strongly affect the MPI-relevant tracer response. Among the 10 nm formulations, Z2 generated stronger absolute harmonic signals than F2 under both conditions and produced the second-highest harmonic amplitudes in the agar phantom. T1-weighted MRI phantom imaging further confirmed concentration-dependent signal attenuation. Z2 and F2 generated more localized hypointense contrast under T1-weighted imaging conditions, while Z1 and F1 generated susceptibility-related signal loss extending beyond the physical boundaries of the inclusions at equivalent nanoparticle concentrations. This spatially extended hypointensity can obscure boundary definition and complicate quantitative signal-intensity analysis. Overall, these findings clarify how core size and Zn substitution regulate the magnetic, biological, MPI-relevant, and MRI-relevant behavior of ferrite nanoparticles and identify Z2 as a promising balanced tracer candidate for dual-modal MPI-MRI imaging.

## Graphical abstract

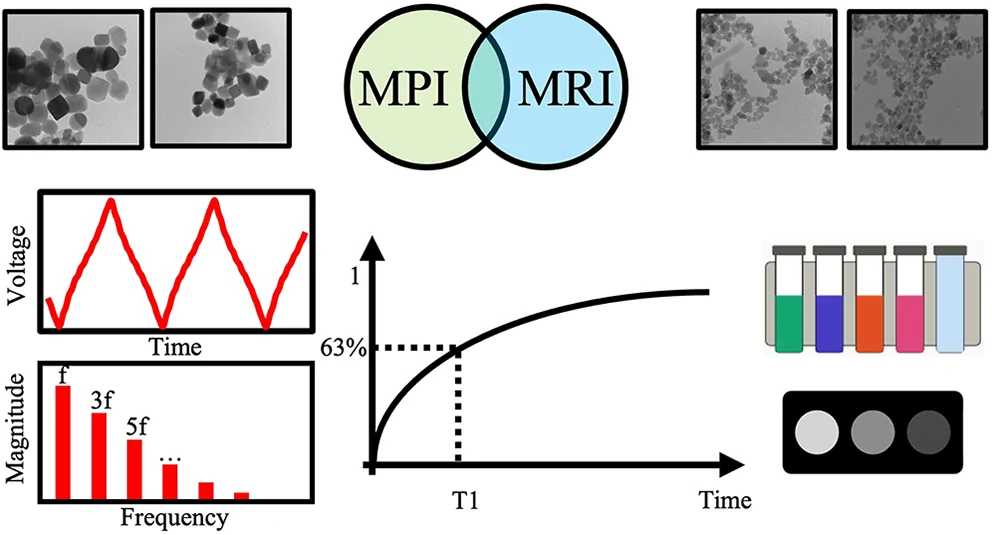

## 1 Introduction

Magnetic particle imaging (MPI) is an emerging tracer-based imaging modality that directly maps the spatial distribution of magnetic nanoparticle (MNP) tracers.^1–4^ First introduced by Gleich and Weizenecker,^5^ MPI exploits the nonlinear magnetization response of superparamagnetic nanoparticles under an alternating magnetic field, producing a signal only from the magnetic tracer rather than from surrounding tissue. Because the MPI signal is positive, background-free, and linearly proportional to the MNP tracer amount, it has attracted increasing interest for quantitative imaging applications such as vascular imaging, cell tracking, tumor imaging, and image-guided therapy.^6–8^ Magnetic resonance imaging (MRI), on the other hand, provides excellent soft tissue contrast and anatomical resolution, but often requires contrast agents to improve sensitivity to specific tissues, lesions, or biological processes.^9–11^ Iron oxide MNPs, especially superparamagnetic iron oxide nanoparticles (SPIONs), shorten the transverse relaxation time of nearby water protons and are commonly used as T2* or T2-weighted negative contrast enhancers. However, increasing nanoparticle concentration can enhance transverse relaxation effects and counteract T1-related signal enhancement, resulting in reduced signal intensity even in T1-weighted images.^12,13^ Clinically used or clinically investigated iron oxide MRI agents have included Feridex/Endorem, Resovist, Combidex/Sinerem, and ferumoxytol;^14–17^ among these, Feridex and Resovist were approved for liver MRI in several regions, while ferumoxytol has been widely investigated and used off-label for MRI because of its long blood-pool residence time and iron-based metabolism.^14,18^ More recently, Ferabright, a ferumoxytol injection, received FDA approval as an iron-based MRI contrast agent for imaging adult patients with known or suspected malignant brain neoplasms, indicating emerging clinical interest in iron oxide-based MRI contrast agents.^19^

In recent years, the complementary strengths of MPI and MRI have motivated increasing interest in dual-modal MPI-MRI imaging.^20–22^ MPI provides highly sensitive and quantitative detection of MNP biodistribution, while MRI provides anatomical context and high-resolution soft-tissue information that MPI alone does not supply. This combination is valuable since SPIONs can generate imaging contrast for both modalities, enabling coregistered anatomical and quantitative tracer information from a single nanoparticle platform.^20,23^ Integrated or sequential MPI-MRI systems have already been explored for *in vivo* cardiovascular imaging, where MRI was used for anatomical localization, and MPI was used for real-time 3D bolus tracking and hemodynamic analysis.^21^ Dual-modal MPI-MRI imaging could benefit several real-world applications, including cardiovascular angiography and perfusion imaging, cancer detection and staging, cell tracking, sentinel lymph node mapping, and image-guided magnetic hyperthermia. In oncology, MPI can quantify the amount and distribution of MNPs in tumors, while MRI can define tumor morphology and surrounding anatomy, which is important for treatment planning, therapeutic monitoring, and evaluating intratumoral delivery uniformity.^20,24^ Prior studies^20,24,25^ have shown that nanoparticle size, composition, magnetic anisotropy, and surface chemistry strongly influence both MRI relaxivity and MPI signal, making rational MNP design central to the development of effective dual-modal contrast agents. In particular, Zn substitution can alter the magnetic properties of iron oxide nanoparticles and has been reported to enhance MPI performance, while particle size and morphology can influence magnetic anisotropy, relaxation behavior, and MRI relaxivity.^26–29^ Importantly, nanoparticle characteristics that favor high MRI contrast do not necessarily result in optimal MPI performance, as the two modalities rely on different magnetic response mechanisms.^30^ Therefore, the four formulations investigated here were selected to systematically compare the effects of core size, composition, and associated magnetic anisotropy, and to determine whether these parameters influence MRI and MPI performance in similar or competing ways.

In this work, we report a systematic evaluation of four lab-synthesized, quasi-cubic MNPs for dual MPI-MRI applications: 30 nm Fe_3_O_4_ nanoparticles (F1), 10 nm Fe_3_O_4_ nanoparticles (F2), 30 nm Zn_0.4_Fe_2.6_O_4_ nanoparticles (Z1), and 10 nm Zn_0.4_Fe_2.6_O_4_ nanoparticles (Z2). By comparing two core sizes and two ferrite compositions, we examine how particle size and Zn substitution regulate crystal structure, morphology, hydrodynamic behavior, surface functional groups, static magnetization behavior, dynamic magnetization response, cytocompatibility, cellular uptake, and T1-weighted MRI signal response. Magnetic particle spectroscopy (MPS) was used as an MPI-relevant screening method to evaluate the dynamic magnetization responses of these nanoparticles at 7.75 kHz. These results establish a direct structure–property-function relationship for Fe_3_O_4_ and Zn-substituted ferrite nanoparticles and identify design considerations for MNP tracers intended for dual MPI-MRI imaging.

## 2 Materials and Methods

### 2.1 Materials

Iron and zinc precursor salts, including ferric chloride hexahydrate (FeCl_3_·6H_2_O, ≥ 98%), ferrous chloride tetrahydrate (FeCl_2_·4H_2_O, ≥98%), and zinc nitrate hexahydrate (Zn­(NO_3_)_2_·6H_2_O, ≥98%), were purchased from Sigma-Aldrich (St. Louis, MO, USA). Reagents used for nanoparticle synthesis, surface modification, and sample preparation included sodium hydroxide (NaOH, pellets, ≥98%), ammonium hydroxide (NH_4_OH), citric acid (≥99.5%), 200-proof ethanol, agar, sodium alginate, phosphate-buffered saline (PBS), and 0.25% trypsin solution, were purchased from Sigma-Aldrich (St. Louis, MO, USA) and Fisher Scientific (Waltham, MA, USA). All water-based solutions were prepared with deionized water. For cell experiments, cells obtained from ATCC (Manassas, VA, USA) were maintained in DMEM supplemented with fetal bovine serum and antibiotics, both purchased from Corning Inc. (Corning, NY, USA). Live/dead staining was performed using a Calcein-AM/EthD-1 kit from Biotium (Fremont, CA, USA). Unless otherwise noted, all chemicals and biological reagents were used directly as supplied.

### 2.2 Synthesis of MNPs

Four MNP formulations were synthesized in this study: 30 nm Fe_3_O_4_ nanoparticles (F1), 10 nm Fe_3_O_4_ nanoparticles (F2), 30 nm Zn_0.4_Fe_2.6_O_4_ nanoparticles (Z1), and 10 nm Zn_0.4_Fe_2.6_O_4_ nanoparticles (Z2). The larger F1 and Z1 nanoparticles were prepared using a modified hydrothermal method based on a previously reported procedure,^31–33^ whereas the smaller F2 and Z2 nanoparticles were synthesized using a modified alkaline coprecipitation method.^34,35^ Citric acid was introduced during synthesis to improve aqueous dispersibility and provide electrostatic stabilization.

F1 and Z1 nanoparticles were prepared through a hydrothermal method designed to promote the growth of larger ferrite nanocrystals. For F1, iron precursor salts were dissolved in deionized water under stirring. For Z1, Zn­(NO_3_)_2_·6H_2_O was added together with the iron precursors at the required stoichiometric ratio to yield Zn_0.4_Fe_2.6_O_4_. The pH of the precursor solution was adjusted to alkaline conditions using NH_4_OH, and the mixture was stirred briefly at room temperature to initiate precipitation. The precipitate was collected by centrifugation and washed several times with deionized water to remove excess ammonium ions and unreacted salts. The washed precipitate was redispersed in deionized water, followed by the addition of citric acid. The suspension was stirred for 30 min to allow citrate molecules to adsorb onto the particle surfaces, improving colloidal stability and limiting uncontrolled aggregation during hydrothermal growth. The resulting suspension was transferred into a Teflon-lined stainless-steel autoclave and subjected to hydrothermal treatment at 195 °C for 16 h to promote the formation of quasi-cubic F1 and Z1 nanoparticles. After the reaction, the autoclave was allowed to cool naturally to room temperature. The nanoparticles were then magnetically collected, washed with deionized water and acetone, and dried under vacuum at 60 °C. The final products were labeled F1 for 30 nm Fe_3_O_4_ and Z1 for 30 nm Zn_0.4_Fe_2.6_O_4_.

F2 and Z2 nanoparticles were synthesized using an alkaline coprecipitation route. For the preparation of F2, FeCl_3_·6H_2_O and FeCl_2_·4H_2_O were dissolved in 30 mL of deionized water under vigorous stirring at 35 °C, with a Fe^3+^/Fe^2+^ molar ratio of 1.9:1. For the synthesis of Z2, Zn­(NO_3_)_2_·6H_2_O was introduced together with the iron precursors at the required molar ratio to obtain the nominal Zn_0.4_Fe_2.6_O_4_ composition. After complete dissolution of the metal precursors, freshly prepared NaOH solution (2 M, 50 mL) was added dropwise under continuous stirring until the solution pH reached approximately 11. The addition of base produced an immediate black precipitate, indicating the formation of ferrite nanoparticles. The reaction mixture was maintained at 35 °C for 1.5 h to allow particle nucleation and growth. Citric acid solution was then added to the suspension, and the mixture was stirred for an additional 30 min to allow citrate adsorption onto the nanoparticle surfaces. The resulting nanoparticles were collected magnetically and washed repeatedly with deionized water and ethanol to remove unreacted precursors, excess base, and loosely bound surface species. The purified particles were dried under vacuum at 60 °C to obtain the final F2 and Z2 nanoparticle powders.

All dried nanoparticle powders were stored at room temperature before further characterization. For solution-phase experiments, including DLS, MPS, cytocompatibility, cellular uptake, and MRI phantom preparation, the nanoparticle powders were redispersed in deionized water or the corresponding experimental medium by vortexing and sonication before use.

### 2.3 Morphology Characterizations

Nanoparticle morphology was examined using transmission electron microscopy (TEM; Hitachi 7650). Dilute nanoparticle suspensions were drop-cast onto carbon-coated copper grids (Sigma-Aldrich, St. Louis, MO, USA) and dried before imaging. Representative TEM images were collected for each nanoparticle formulation to evaluate particle shape, dispersity, and aggregation state. Particle core sizes were quantified from TEM images using ImageJ software by measuring individual particle diameters.

### 2.4 Hydrodynamic Size Characterizations

The colloidal properties of the nanoparticles were characterized by dynamic light scattering (DLS) using a Microtrac Zetatrac system, Model NPA152. Nanoparticles were dispersed in deionized water at a final concentration of 500 μg/mL and sonicated for 30 min before the measurement. The hydrodynamic diameter was measured to evaluate their dispersion state in aqueous solution. These measurements were used to assess colloidal stability and to compare the influence of core size and Zn substitution on the surface electrostatic properties of nanoparticle formulations.

### 2.5 Crystalline Structure Characterizations

The crystalline phase and structural purity of the nanoparticles were analyzed by powder X-ray diffraction (XRD) using a Rigaku MiniFlex 6G diffractometer with Cu Kα radiation (λ = 1.5406 Å). Dried nanoparticle powders were placed on the sample holder and scanned over a 2θ range of 20–80° using a step size of 0.1° and a 1.25° slit. The diffraction patterns were used to confirm the formation of the spinel ferrite structure and to compare the crystallographic features of Fe_3_O_4_ and Zn-substituted ferrite nanoparticles.

### 2.6 Functional Group Characterizations

Fourier-transform infrared spectroscopy (FTIR) was performed to identify surface functional groups and verify the presence of surface coatings or ligands on the nanoparticles. Dried nanoparticles were analyzed over the wavenumber range of 400–4000 cm^–1^, and characteristic absorption bands were assigned to metal–oxygen vibrations from the ferrite core and organic functional groups associated with surface ligands. The FTIR spectra were compared among F1, F2, Z1, and Z2 to evaluate whether the surface chemistry was preserved across different particle sizes and compositions.

### 2.7 Static Magnetization Characterizations

Room-temperature, static magnetic properties were measured using a Physical Property Measurement System (PPMS; Quantum Design). Dried nanoparticles, consisting of 1.4 mg of Z1, 3.0 mg each of F1 and F2, and 3.4 mg of Z2, were wrapped in Parafilm and placed in the sample holder prior to analysis. Magnetization curves were collected under applied magnetic fields up to 1.5 T. The resulting static M-H loops were used to determine saturation magnetization (*M*_s_), coercivity (*H*_c_), and remanent magnetization (*M*_r_). These parameters were compared across the four nanoparticle formulations to evaluate the effects of particle size and Zn substitution on static magnetic behavior. The measured magnetic properties were further used to interpret differences in dynamic magnetization response and MRI contrast performance.

### 2.8 Dynamic Magnetization Characterizations

The dynamic magnetization responses of the nanoparticles were characterized using a custom-built MPS platform,^36–38^ as shown in Figure 1A. Because MPI contrast originates from the nonlinear magnetization responses of MNPs under alternating magnetic fields (i.e., excitation fields), MPS provides a direct spectroscopic method to compare MPI-relevant tracer performance.^39–41^

**Figure 1:**
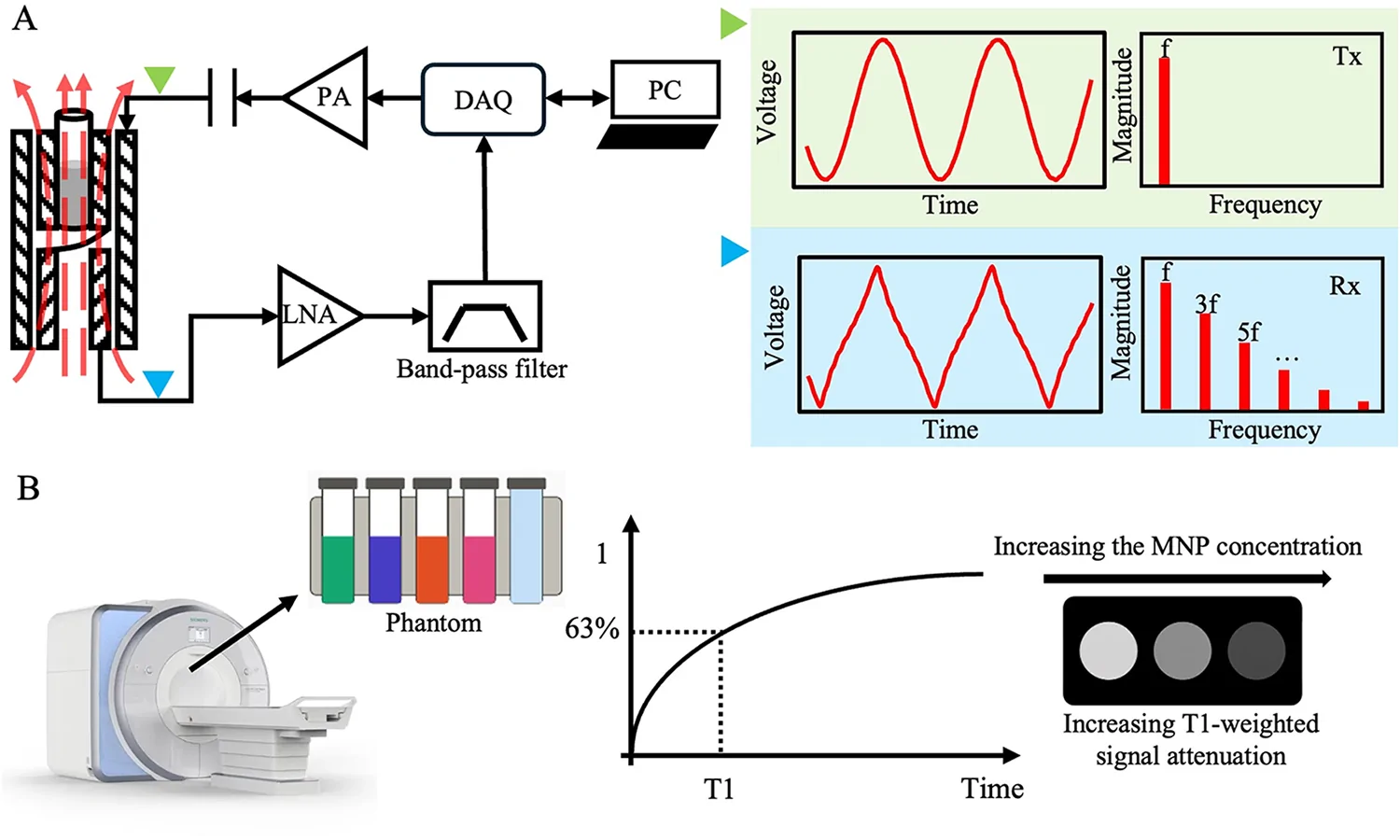
(A) Schematics of signal flows in the custom-built MPS platform. Example input excitation field waveforms in the transmission coil (Tx) and dynamic magnetization responses induced voltage waveforms in the receive coils (Rx). (B) Schematic of the T1-weighted MRI characterization on the MNP phantoms.

For aqueous MPS measurements, dried nanoparticles were dispersed in deionized water at a concentration of 6 mg/mL, and 100 μL of each suspension was loaded into the MPS sample holder. For agar phantom preparation, a 2% (w/v) agar solution was prepared in deionized water under heating. The dried nanoparticles were separately dispersed in deionized water at 12 mg/mL, and 50 μL of each MNP suspension was mixed with 50 μL of the agar solution, resulting in a final MNP concentration of 6 mg/mL and a final agar concentration of 1% (w/v). MNP phantoms (water and agar phantoms) were exposed to a 7.75 kHz sinusoidal excitation field with peak amplitudes of 5, 10, 15, and 20 mT. These field amplitudes were selected to drive the nanoparticles into their nonlinear magnetization regime. Because the MPS signal originates from the same nonlinear magnetization dynamics that generate MPI contrast,^42–44^ these measurements were used to compare the MPI suitability of F1, F2, Z1, and Z2. Differences in harmonic intensity and spectral decay were analyzed to determine how core size and Zn substitution affect the dynamic magnetization behavior of the nanoparticles under MPI-relevant excitation conditions.

### 2.9 Cytotoxicity Test

The cytocompatibility of the MNP formulations was evaluated using SKOV3 ovarian cancer cells and MCF7 breast cancer cells. Cells were seeded in culture plates and maintained under standard cell culture conditions until they reached suitable confluency for nanoparticle treatment. F1, F2, Z1, and Z2 nanoparticle suspensions were added to the cell culture medium at final concentrations of 100 and 500 μg/mL, followed by incubation for 24 h. After nanoparticle exposure, the culture medium was removed, and the cells were gently washed with PBS to remove loosely attached nanoparticles. Cell viability was then assessed using a fluorescence-based Live/Dead assay containing Calcein-AM and EthD-1 according to the manufacturer’s protocol. Calcein-AM was used to label metabolically active live cells with green fluorescence, while EthD-1 was used to identify membrane-compromised dead cells with red fluorescence. Fluorescence images were acquired using an inverted fluorescence microscope under identical imaging conditions for comparison among treatment groups. The relative abundance of green and red fluorescence was used to assess the short-term cytocompatibility of each nanoparticle formulation at the tested concentrations.

### 2.10 Cellular Association and Uptake Test

Cellular association and uptake of the MNP formulations were evaluated qualitatively in SKOV3 ovarian cancer cells using bright-field microscopy. SKOV3 cells were seeded in 6-well plates at an appropriate density and cultured under standard conditions to allow cell attachment before nanoparticle treatment. F1, F2, Z1, and Z2 nanoparticles were dispersed in complete cell culture medium and added to the cells at final concentrations of 100 and 500 μg/mL. The cells were then incubated with the nanoparticles for 24 h. After incubation, the nanoparticle-containing medium was removed, and the cells were gently washed with PBS to remove loosely bound or free nanoparticles. The cells were then fixed with 4% paraformaldehyde for 15 min at room temperature. Bright-field images were acquired using an EVOS M5000 Imaging System (AMF5000SV; Thermo Fisher Scientific, Waltham, MA, USA) to visualize cell-associated nanoparticle accumulation. Dark contrast associated with the cell bodies and pericellular regions was used as a qualitative indicator of nanoparticle association and/or uptake. Because bright-field microscopy does not distinguish surface-bound nanoparticles from fully internalized nanoparticles, the observed signal was interpreted as cell-associated nanoparticle accumulation in this paper.

### 2.11 T1-Weighted MRI Characterizations

T1-weighted MRI signal response of the four MNP formulations was examined using agar-based phantoms (Figure 1B). For MRI phantom preparation, a 2% (w/v) agar solution was prepared in deionized water under heating. MNP suspensions were prepared separately at concentrations twice the desired final values and then mixed with the agar solution at a 1:1 volume ratio. Specifically, 2.5 mL of each MNP suspension was combined with 2.5 mL of the agar solution to yield a final volume of 5 mL, with final MNP concentrations of 100 and 500 μg/mL and a final agar concentration of 1% (w/v). T1-weighted images were acquired on a clinical 3.0 T MRI scanner (MAGNETOM Skyra, Siemens Healthcare, Erlangen, Germany) using a 2D turbo spin–echo (TSE) sequence with a repetition time (TR) of 753 ms, an effective echo time (TE_eff_) of 11 ms, an echo train length (ETL) of 3, a field of view (FOV) of 160 × 240 mm, a slice thickness of 5 mm, and two averages. The resulting images were used to compare the size- and Zn-substitution-dependent signal attenuation under T1-weighted imaging conditions produced by F1, F2, Z1, and Z2.

## 3 Results and Discussion

### 3.1 Morphology, Crystal Structure, and Hydrodynamic Size Characterizations of MNPs

The morphology and size characteristics of the four MNP formulations were first examined by TEM. Figure 2A shows the 30 nm quasi-cubic Fe_3_O_4_ sample, F1, which displays predominantly particles with faceted edges, consistent with crystal growth under hydrothermal conditions. Although some particle overlap is observed, the individual cores show a larger size and more defined cubic geometry than the coprecipitated samples. Figure 2B shows the 10 nm quasi-cubic Fe_3_O_4_ sample, F2, prepared by coprecipitation. Compared with F1, F2 consists of smaller particles with less sharply defined facets and a quasi-cubic morphology, indicating that the lower-temperature coprecipitation process favors rapid nucleation and limited crystal growth. The same synthesis-dependent morphological trend was observed for the Zn-substituted ferrites. Figure 2C shows Z1, the 30 nm quasi-cubic Zn_0.4_Fe_2.6_O_4_ sample synthesized by the hydrothermal method. Z1 exhibits a more cubic morphology like F1, indicating that Zn substitution does not prevent the formation of faceted ferrite nanocrystals under hydrothermal growth conditions. Figure 2D shows Z2, the 10 nm quasi-cubic Zn_0.4_Fe_2.6_O_4_ sample synthesized by coprecipitation. Z2 is composed of smaller quasi-cubic nanoparticles, like F2. Quantitative TEM analysis (insets of Figure 2A–D) yielded mean core sizes of 29.9 ± 10.3 nm for F1, 10.5 ± 2.3 nm for F2, 30.2 ± 12.3 nm for Z1, and 9.5 ± 2.0 nm for Z2. These mean core sizes were consistent with the nominal 10 and 30 nm designations used in our work.

**Figure 2:**
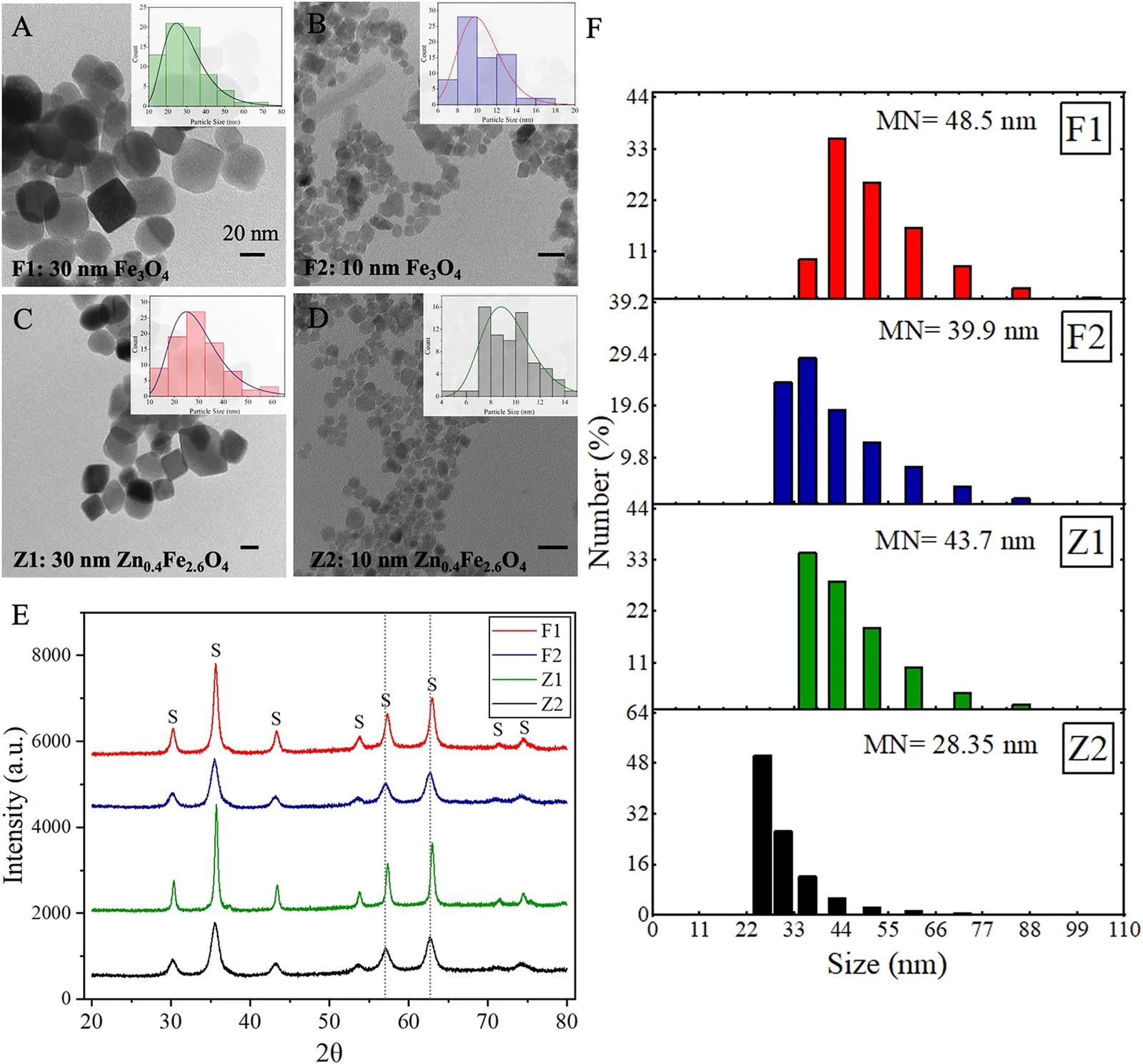
Morphology, crystal structure, and hydrodynamic size distributions of F1, F2, Z1, and Z2 nanoparticles. Representative TEM images of (A) 30 nm quasi-cubic Fe_3_O_4_ nanoparticles (F1), (B) 10 nm quasi-cubic Fe_3_O_4_ nanoparticles (F2), (C) 30 nm quasi-cubic Zn_0.4_Fe_2.6_O_4_ nanoparticles (Z1), and (D) 10 nm quasi-cubic Zn_0.4_Fe_2.6_O_4_ nanoparticles (Z2). Scale bars: 20 nm. (E) Powder XRD patterns of the four MNP formulations, confirming the spinel ferrite crystal structure. Peaks labeled “S” correspond to characteristic spinel reflections. (F) DLS-derived hydrodynamic size distributions of F1, F2, Z1, and Z2 dispersed in deionized water. MN represents the number-weighted mean diameter shown in each panel.

The crystalline phases of the nanoparticles were characterized by powder XRD, as shown in Figure 2E. All four samples exhibit diffraction peaks that can be assigned to a spinel ferrite structure, and no obvious secondary crystalline phases are detected within the measured 2θ range. The experimental patterns were matched with the corresponding crystallographic reference patterns: F1 (reference code: 96–900–5840), F2 (reference code: 96–900–5839), Z1 (reference code: 96–900–6898), and Z2 (reference code: 96–900–8696). The peaks labeled “S” correspond to the characteristic spinel reflections, confirming successful formation of Fe_3_O_4_ and Zn-substituted ferrite nanoparticles. The diffraction peaks of the hydrothermally synthesized samples (F1 and Z1) are generally sharper than those of the smaller coprecipitated samples (F2 and Z2), which is consistent with their larger crystalline domains. Furthermore, the Zn-containing samples, Z1 and Z2, also retain the spinel phase, indicating that Zn was incorporated without generating a separate Zn-containing impurity phase detectable by XRD. The Zn^2+^ substitution alters the lattice parameter because Zn^2+^ has a different ionic radius and site preference than Fe^2+^/Fe^3+^, and it can also affect Fe^2+^/Fe^3+^ distribution between tetrahedral and octahedral sites.^31,45,46^ Therefore, subtle shifts in peak position (marked as dashed lines in Figure 2E) are consistent with Zn-induced lattice modification and cation redistribution.

The hydrodynamic size distributions of the nanoparticles dispersed in deionized water are summarized in Figure 2F. The DLS-derived mean hydrodynamic diameters are 48.5 nm for F1, 39.9 nm for F2, 43.7 nm for Z1, and 28.35 nm for Z2. These hydrodynamic diameters are larger than the nominal TEM core sizes because DLS measures the hydrated particle size in solution, including the surface citrate layer, solvation shell, and any small aggregates present in the dispersion. For the larger hydrothermal samples, F1 and Z1, the hydrodynamic diameters were moderately larger than their TEM-derived core sizes, which is consistent with contributions from the citrate-associated hydration layer and possible limited particle association in aqueous suspension. In contrast, the smaller coprecipitated samples exhibited larger relative increases in hydrodynamic size, particularly F2, suggesting a greater degree of particle association or clustering in deionized water. Z2 exhibited a smaller hydrodynamic diameter than F2, suggesting comparatively better aqueous dispersion or less extensive particle association under the same measurement conditions.

### 3.2 Physicochemical Characterization of MNPs

The static magnetization properties of the four MNP formulations were evaluated by room-temperature M-H measurements using PPMS. As shown in Figure 3A, all samples exhibit ferrimagnetic/superparamagnetic-like magnetization curves with rapid magnetization reversal near zero field and approach saturation under high applied fields. The saturation magnetization values fall within approximately 60–80 emu/g, confirming that all four formulations retain strong magnetization responses suitable for magnetic imaging and sensing applications. Among the Fe_3_O_4_ samples, the larger F1 (30 nm) shows higher magnetization than the smaller F2 (10 nm), consistent with the reduced surface spin disorder and larger magnetic volume expected for larger particles.^47–50^ Z2 (10 nm) exhibits comparable and slightly higher magnetization than Z1 (30 nm). In addition, Zn substitution enhances the magnetization relative to the corresponding Fe_3_O_4_ samples, with Z1 and Z2 showing higher magnetization than F1 and F2, respectively. This enhancement is consistent with partial Zn^2+^ incorporation into the spinel ferrite lattice, which can modify cation distribution between tetrahedral and octahedral sites and increase the net magnetic moment. The inset of Figure 3A shows the low-field region of the M-H loops, and it is observed that all four samples have small coercive fields. The estimated coercivities are approximately 2.5 mT for F1, 1.0 mT for F2, 0.1 mT for Z1, and 0.3 mT for Z2. The relatively small coercivity values indicate that the nanoparticles can respond readily to alternating magnetic fields, which is important for MPI-relevant dynamic magnetization. The lower coercivity of the Zn-substituted samples, especially Z1, suggests that Zn incorporation may soften the magnetic response, while still maintaining strong saturation magnetization. This combination of high magnetization and low coercivity is favorable for generating strong nonlinear magnetic responses under MPS/MPI excitation.

**Figure 3:**
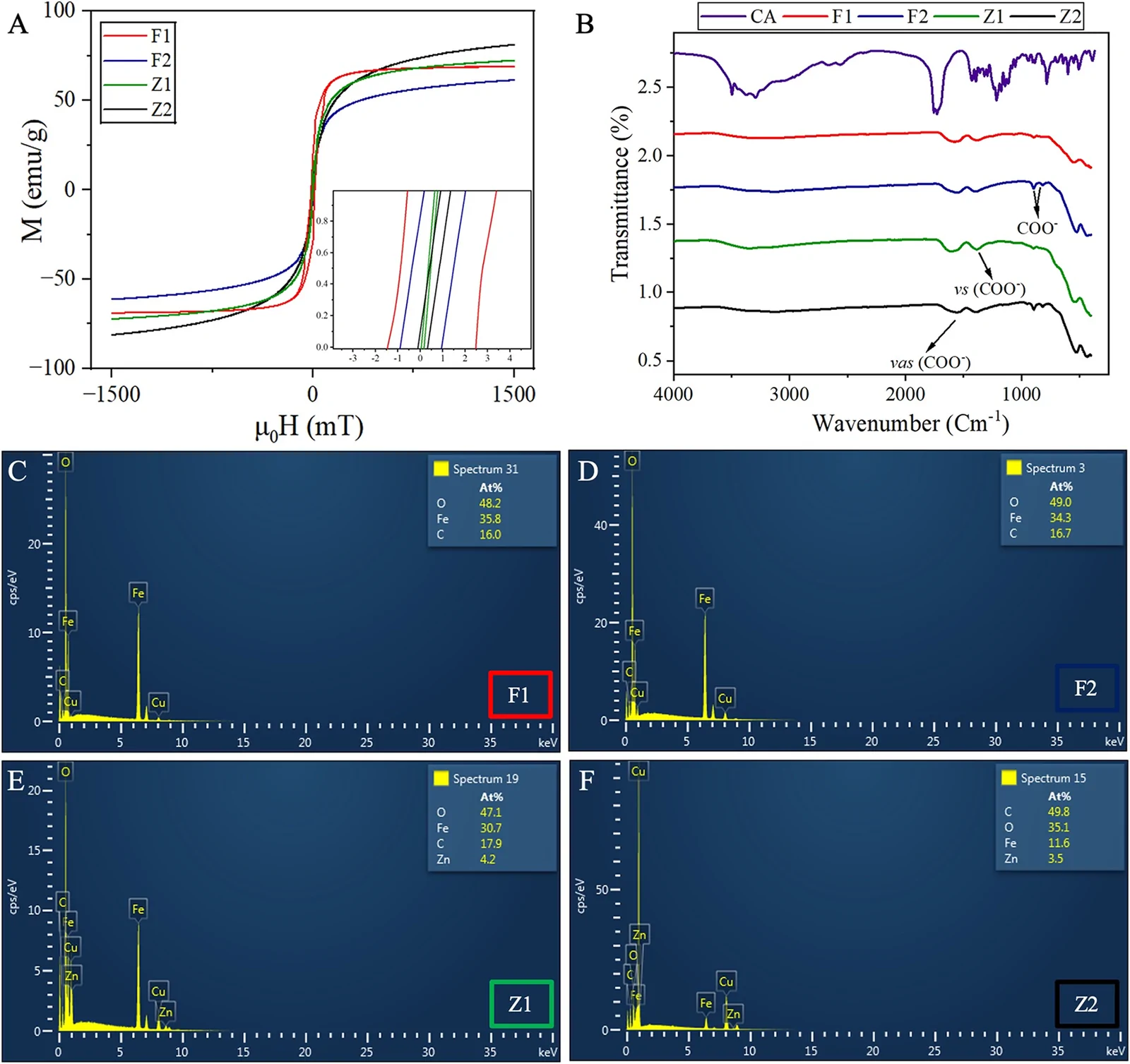
Static magnetization behaviors, surface chemistry, and elemental composition of F1, F2, Z1, and Z2 nanoparticles. (A) Room-temperature M-H curves of F1, F2, Z1, and Z2 measured under an applied magnetic field from −1.5 to +1.5 T; the inset shows the low-field region used to compare coercivities. (B) FTIR spectra of citric acid and citrate-functionalized F1, F2, Z1, and Z2 nanoparticles, showing characteristic surface functional groups associated with citrate coordination. (C)-(F) EDX spectra of (C) F1, (D) F2, (E) Z1, and (F) Z2, confirming Fe and O signals in the Fe_3_O_4_ formulations and additional Zn signals in the Zn_0.4_Fe_2.6_O_4_ formulations.

The surface chemistry of the nanoparticles was examined by FTIR spectroscopy, as shown in Figure 3B. The spectrum of citric acid shows characteristic vibrational features associated with carboxyl and hydroxyl-containing groups. After nanoparticle functionalization, F1, F2, Z1, and Z2 show absorption bands assigned to citrate-related carboxylate vibrations, including asymmetric and symmetric COO^–^ stretching bands near 1585 and 1385 cm^–1^, respectively, as well as additional citrate-related COO^–^ vibrations near 900 and 826 cm^–1^, consistent with previously reported FTIR spectra of citrate-coated iron oxide nanoparticles.^51–53^ The presence of these bands indicates successful citrate coordination on the nanoparticle surfaces. The citrate coating is important for biomedical applications because it improves aqueous dispersibility and provides negatively charged functional groups that help stabilize the particles in suspension.

Elemental analysis by EDX further supports the composition of the synthesized nanoparticles. Figure 3C shows the EDX spectrum of F1, where Fe and O signals dominate, consistent with the Fe_3_O_4_ core composition. A carbon signal is also detected, which may come from the organic surface species and the TEM grid. The copper peak arises from the copper TEM grid. For F2, the EDX spectrum in Figure 3D similarly shows Fe and O as the principal nanoparticle elements, confirming the iron oxide composition of the smaller quasi-cubic Fe_3_O_4_ formulation. As with F1, the carbon and copper signals are also observed. The Zn-substituted samples show additional Zn signals in the EDX spectra. Figure 3E confirms the presence of Fe, O, and Zn in Z1, consistent with the intended Zn_0.4_Fe_2.6_O_4_ composition. The detected Zn signal indicates successful incorporation of zinc into the ferrite nanoparticle formulation. Figure 3F shows a similar Fe–O–Zn elemental profile for Z2, confirming that Zn is also present in the smaller quasi-cubic Zn-substituted ferrite nanoparticles. The comparable elemental signatures of Z1 and Z2 support the successful synthesis of Zn-containing ferrite nanoparticles by both hydrothermal and coprecipitation routes.

### 3.3 Cytotoxicity Characterization of MNPs

The short-term cytocompatibility of the four MNP formulations was evaluated using a fluorescence-based Live/Dead assay after 24 h incubation with SKOV3 ovarian cancer cells and MCF7 breast cancer cells. For SKOV3 cells, representative fluorescence images are shown in Figure 4A-**I**. Cells treated with F1 at 100 and 500 μg/mL maintained strong green fluorescence with only occasional, red-stained cells (Figure 4A,B). A similar trend was observed for F2-treated cells (Figure 4C,D), where most cells remained viable even at 500 μg/mL. The Zn-substituted formulations also showed good cytocompatibility. Z1-treated SKOV3 cells retained widespread green fluorescence at both concentrations (Figure 4E,F), and Z2-treated cells also showed predominantly live-cell staining with minimal red fluorescence (Figure 4G,H). For reference, Figure 4I shows SKOV3 cells with no MNPs added. Although local differences in cell density and clustering are visible among images, there is no obvious concentration-dependent increase in dead-cell staining from 100 to 500 μg/mL for any of the four nanoparticle formulations. These results suggest that both Fe_3_O_4_ and Zn_0.4_Fe_2.6_O_4_ nanoparticles are well tolerated by SKOV3 cells over the tested concentration range.

**Figure 4:**
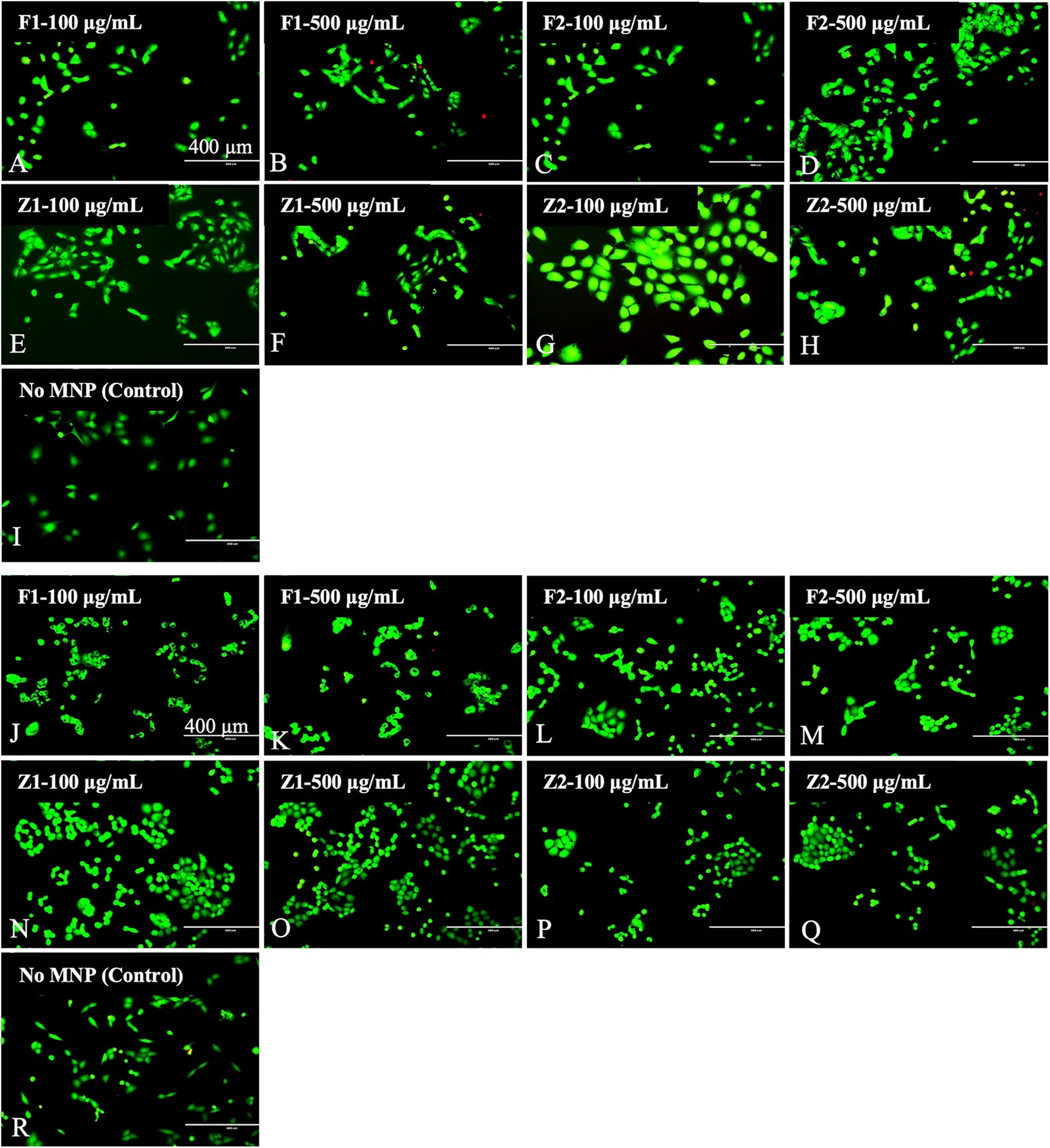
Fluorescence-based Live/Dead cell viability assay of (A–I) SKOV3 ovarian cancer cells and (J–R) MCF7 breast cancer cells after 24 h incubation with MNPs. (I) and (R) are control groups without MNPs. Green fluorescence indicates live cells, and red fluorescence indicates dead cells. Predominantly green fluorescence indicates high cell viability with minimal cytotoxicity. Scale bars: 400 μm.

The same Live/Dead assay was performed using MCF7 breast cancer cells, as shown in Figure 4J–R. Across all treatment groups, MCF7 cells exhibited predominantly green Calcein-AM fluorescence, with no obvious red EthD-1 fluorescence observed after 24 h incubation. Cells treated with F1 at 100 and 500 μg/mL retained strong live-cell staining and preserved cell morphology (Figure 4J,K). Similar results were observed for F2-treated cells (Figure 4L,M), indicating that decreasing the Fe_3_O_4_ core size did not introduce detectable acute cytotoxicity under the tested conditions. The Zn-substituted formulations also showed excellent compatibility with MCF7 cells, as both Z1 (Figure 4N,O) and Z2 (Figure 4P,Q) produced uniformly green fluorescence at both nanoparticle concentrations. The absence of visible red fluorescence suggests that these MNP formulations did not induce membrane-compromising cell death in MCF7 cells after 24 h exposure, even at the higher concentration of 500 μg/mL. For reference, Figure 4R shows MCF7 cells with no MNPs added. Compared with the SKOV3 images in Figure 4A–H, the MCF7 images in Figure 4J–Q qualitatively suggest at least comparable, and possibly stronger, short-term tolerance to the MNP treatments.

It is worth noting that the use of two different cancer cell lines in this paper strengthens the biological relevance of the cytocompatibility assessment. SKOV3 and MCF7 cells differ in tissue origin and cellular phenotype, yet both maintained high viability after exposure to all four MNP formulations. This suggests that the observed compatibility is not limited to a single cell type. The lack of obvious toxicity at 500 μg/mL is encouraging because many reported *in vitro* MNP cell-labeling protocols achieve detectable MRI- or MPI-relevant labeling at lower iron concentrations,^39,54–57^ commonly in the range of ∼3–300 μg Fe/mL, although some ferumoxytol-based protocols use concentrations up to 500 μg Fe/mL. These results support the potential use of F1, F2, Z1, and Z2 as magnetic tracers for biological imaging studies.

### 3.4 Cellular Association and Uptake Behavior of MNPs

The cellular association and uptake behavior of the four MNP formulations were evaluated in SKOV3 ovarian cancer cells after 24 h incubation at 100 and 500 μg/mL. The bright-field images show dark nanoparticle-associated contrast on or around the cells, allowing qualitative comparison of cell-associated MNP accumulation among different formulations and concentrations. For F1, visible nanoparticle deposition was observed after incubation at both 100 and 500 μg/mL. At 100 μg/mL, F1-treated cells showed clear dark contrast associated with the cell bodies and pericellular regions (Figure 5A,B). Increasing the concentration to 500 μg/mL led to stronger and more extensive dark contrast around SKOV3 cells (Figure 5C,D), indicating a concentration-dependent increase in cell-associated nanoparticle accumulation. The appearance of dense particle clusters near the cell boundaries suggests that F1 nanoparticles may be adsorbed onto the cell surface and/or internalized by the cells after 24 h incubation. A similar concentration-dependent trend was observed for Z1. At 100 μg/mL, Z1 produced detectable but relatively moderate dark contrast around MCF7 cells (Figure 5E,F). At 500 μg/mL, the cell-associated contrast became more pronounced, with larger dark aggregates appearing near cell clusters and along cell boundaries (Figure 5G,H). Compared with the 100 μg/mL condition, the 500 μg/mL Z1-treated cells showed a clear increase in nanoparticle accumulation, suggesting that the larger Zn-substituted ferrite nanoparticles can effectively associate with SKOV3 cells under higher exposure conditions.

**Figure 5:**
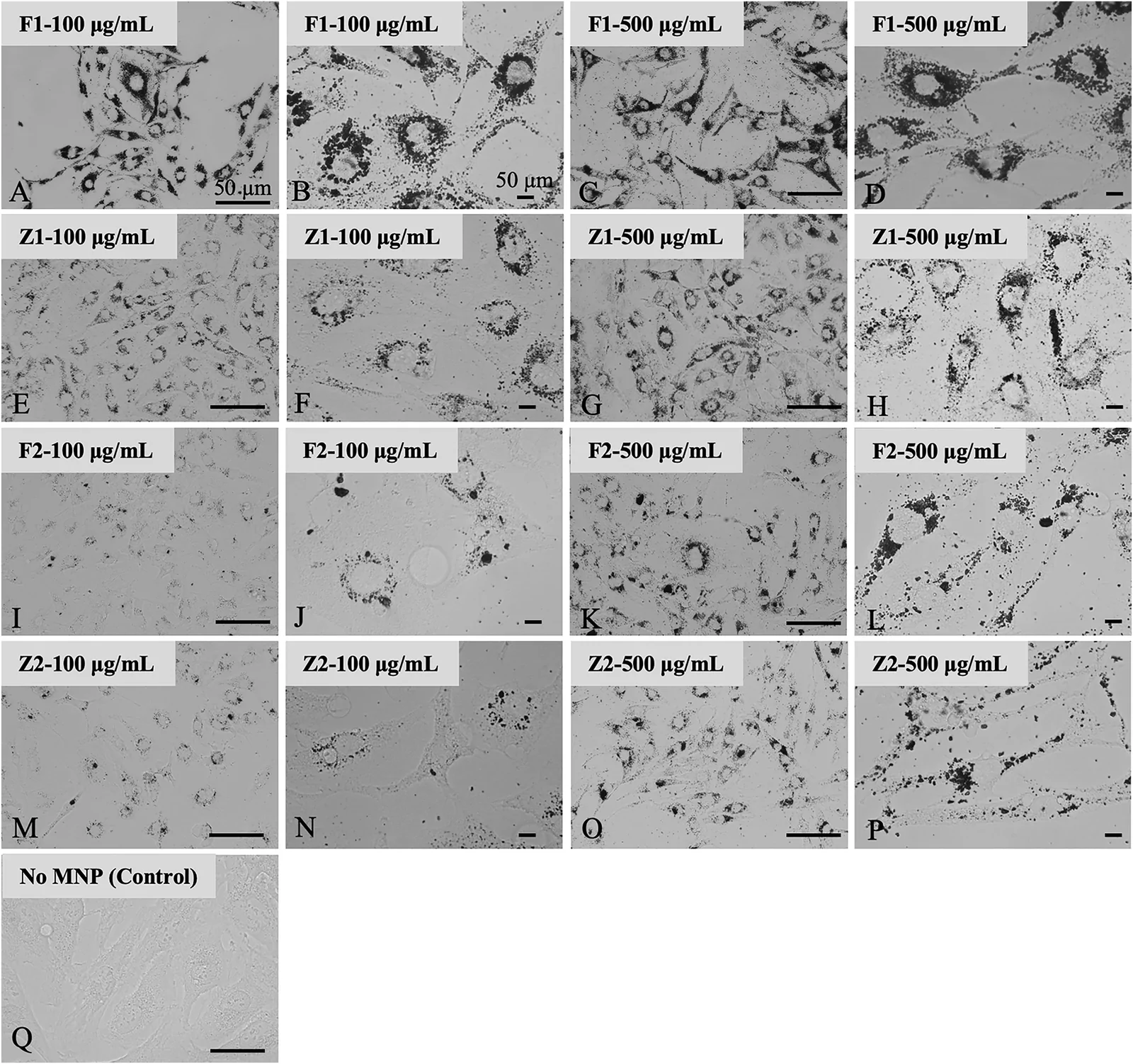
Cellular association and uptake of MNPs in SKOV3 breast cancer cells. Representative bright-field microscopy images of SKOV3 cells after 24 h incubation with F1 (A)-(D), Z1 (E)-(H), F2 (I)-(L), and Z2 (M)-(P) nanoparticles at 100 μg/mL and 500 μg/mL. Panels (A), (B), (E), (F), (I), (J), (M), (N) show cells treated at 100 μg/mL, panels (C), (D), (G), (H), (K), (L), (O), (P) show cells treated at 500 μg/mL, and panel (Q) shows cells without addition of MNPs. Dark contrast indicates cell-associated nanoparticle accumulation. Scale bars: 50 μm.

The 10 nm Fe_3_O_4_ formulation, F2, showed weaker uptake-related contrast than the 30 nm formulations. At 100 μg/mL, only limited dark contrast was observed around SKOV3 cells (Figure 5I,J), suggesting relatively low cell-associated accumulation under this condition. At 500 μg/mL, F2-treated cells showed increased nanoparticle contrast compared with the lower concentration (Figure 5K,L), but the overall accumulation appeared less extensive than that observed for F1 and Z1. This difference suggests that smaller Fe_3_O_4_ nanoparticles may have reduced cell association or weaker local contrast per particle cluster under the same incubation conditions. Z2 also showed concentration-dependent accumulation. At 100 μg/mL, Z2-treated cells displayed relatively sparse nanoparticle-associated contrast (Figure 5M,N). At 500 μg/mL, a more visible dark contrast appeared around the cells and within cell clusters (Figure 5O,P), indicating enhanced cell-associated accumulation at the higher dose. Like F2, however, the 10 nm Z2 particles appeared to produce less extensive dark contrast than the larger 30 nm formulations. This suggests that core size may play an important role in SKOV3 cell association, with the 30 nm particles showing stronger apparent uptake than the 10 nm particles under the tested conditions.

For reference, a bright-field microscopy image of SKOV3 cells without the treatment of MNPs (Figure 5Q) is added. Our results indicate that all four MNP formulations can associate with SKOV3 cells after 24 h incubation, but the degree of uptake depends on both concentration and nanoparticle structure. Increasing the incubation concentration from 100 to 500 μg/mL generally increased the amount of visible cell-associated nanoparticle contrast for each formulation. Among the four samples, F1 and Z1 showed (Figure 5A–H) stronger dark contrast than F2 and Z2 (Figure 5I–P), suggesting that the 30 nm particles have higher cell association than the 10 nm particles. This size-dependent trend may indicate more efficient membrane interaction, sedimentation-assisted contact, or endocytic uptake of larger particles.

### 3.5 MPS Characterization of MNPs: Evaluation of MPI Performance

In this work, MPS was used to evaluate the dynamic magnetization behaviors of F1, F2, Z1, and Z2 under MPI-relevant excitation conditions. Herein, the four MNP formulations were first measured in deionized water as a freely dispersed, low-viscosity reference state, in which both Brownian rotation and Néel relaxation can contribute to the dynamic magnetization response.^58,59^ The particles were then embedded in an agar matrix to restrict physical rotation and suppress the Brownian contribution, providing a simplified model of biologically constrained nanoparticles, such as particles immobilized after cell-surface binding, extracellular matrix trapping, or cellular internalization.^60,61^

#### 3.5.1 Dynamic Magnetizations of MNPs in Water Phantoms

Figure 6 summarizes the MPS responses of the water phantoms under 7.75 kHz excitation at field amplitudes of 10, 15, and 20 mT. The time-domain waveforms from the receive coil are shown in Figure 6A,C,E for 10, 15, and 20 mT, respectively. For all four water phantoms, the induced voltage increased as the excitation field amplitude increased, indicating that stronger excitation fields produced larger dynamic magnetization changes. Among the four formulations, Z1 generated the highest voltage response under all three field amplitudes, whereas F1, F2, and Z2 produced lower and more comparable signals. This result suggests that the 30 nm quasi-cubic Zn_0.4_Fe_2.6_O_4_ nanoparticles have the strongest dynamic magnetization response in the freely dispersed state. Although Z2 did not reach the high absolute signal level of Z1, its time-domain response was comparable to or higher than the undoped 10 nm Fe_3_O_4_ sample F2, indicating that Zn substitution still improves the dynamic magnetic response within the smaller 10 nm particle size regime.

**Figure 6:**
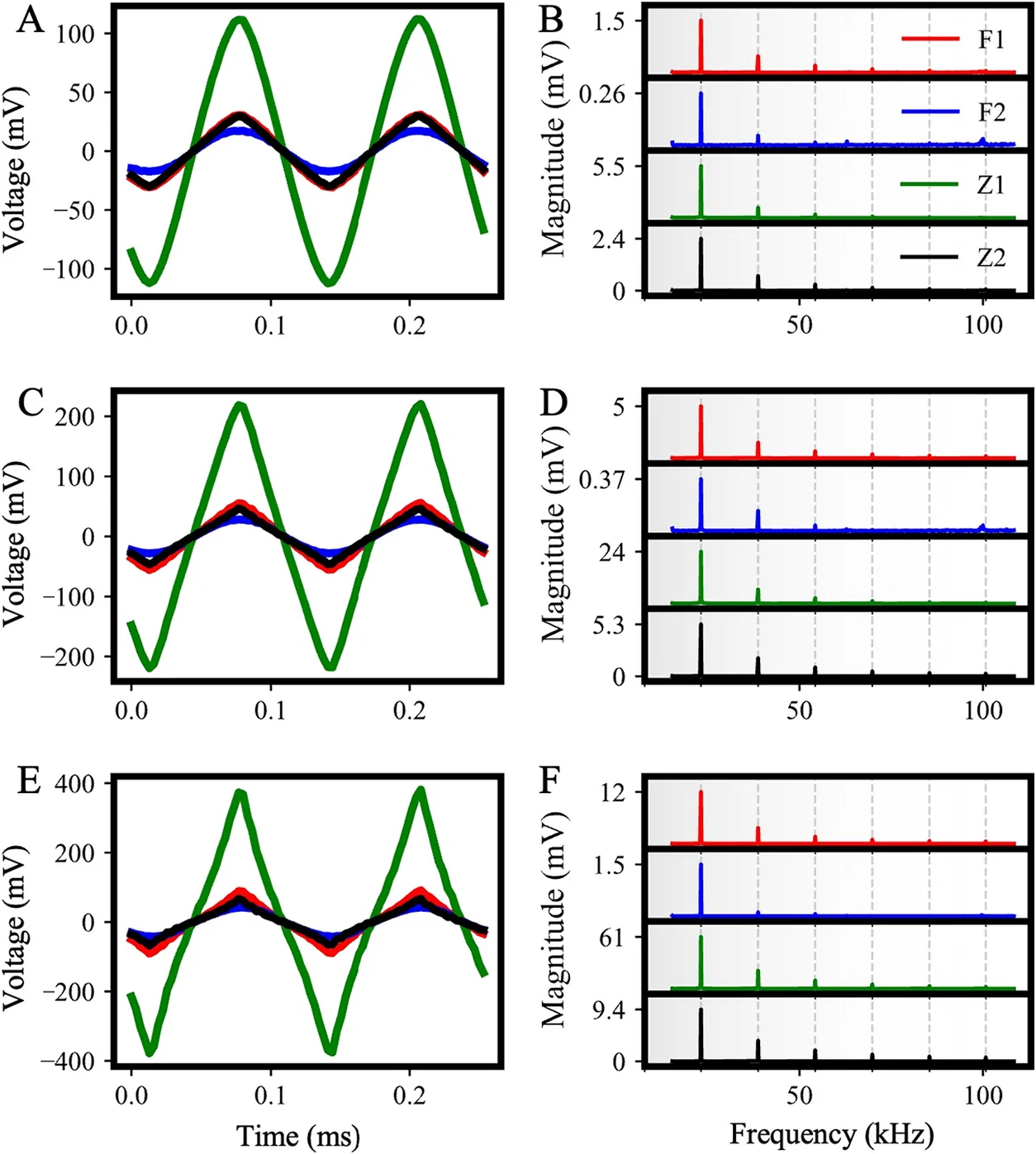
MPS characterization of MNP formulations dispersed in deionized water. The excitation field is set at 7.75 kHz, and the amplitude is set at (A, B) 10 mT, (C, D) 15 mT, and (E, F) 20 mT, respectively. (A), (C), and (E) are the time-domain induced voltage signals collected from the receive coils. (B), (D), and (F) are the 3rd to 13th harmonic spectra; the amplitude of the 3rd component is labeled on the *y*-axis of each curve.

The frequency-domain spectra in Figures 6B,D,F further indicate the nonlinear magnetization responses of these MNPs. After removal of the fundamental excitation frequency component, the higher odd harmonics, including the third, fifth, seventh, and higher orders, were analyzed because these harmonics are the signal components most relevant to MPI detection. At 10 mT (Figure 6B), 15 mT (Figure 6D), and 20 mT (Figure 6F), the harmonic amplitudes decreased with increasing harmonic order, which is expected for MNP tracers under sinusoidal excitation. Z1 again produced the strongest harmonic signals across the measured frequency window, especially at the lower odd harmonics. Since higher harmonic generation is essential for MPI image formation,^5,62–65^ these results identify Z1 as the most promising MPI tracer among the four MNP formulations when the nanoparticles are freely suspended in water. Z2 also generated clear odd harmonic signals across the tested field amplitudes, with higher-order harmonic amplitudes that were generally stronger than or comparable to those of F1 and F2. In addition, the harmonic spectrum of Z2 exhibited the slowest decay with increasing harmonic order among the four MNP formulations, indicating that the 10 nm Zn-substituted ferrite nanoparticles preserve higher-frequency nonlinear magnetic responses more effectively under these excitation conditions. This is important because Z2 later shows more localized signal attenuation under T1-weighted imaging conditions, making its measurable harmonic response relevant for dual-modal tracer selection rather than single-modality MPI optimization alone.

#### 3.5.2 Dynamic Magnetizations of MNPs in Agar Phantoms

In water phantoms, the dynamic response of MNPs can involve both Néel relaxation, where the magnetic moment rotates within the particle, and Brownian relaxation, where the whole particle physically rotates.^66–69^ In agar phantoms, Brownian motion is strongly restricted, so the measured signal becomes dominated by Néel relaxation and any limited local mobility allowed by the agar.^68,70–73^ This comparison is important for biological MPI applications since nanoparticles are often partially or fully immobilized after interacting with proteins, cell membranes, extracellular matrix, or intracellular compartments.

As shown in Figure 7, the agar phantoms showed distinct changes in their MPS responses compared with the water phantoms. The time-domain voltages in Figure 7A, C,E correspond to 10, 15, and 20 mT excitation field strengths, respectively. Compared with Figure 6A,C,E, all formulations showed reduced voltage amplitudes after agar embedding, indicating that immobilization weakens the dynamic magnetization response detected by the receive coils. This reduction is consistent with the suppression of Brownian relaxation. When physical particle rotation is blocked, particles that rely strongly on Brownian motion in solution can no longer follow the alternating magnetic field as effectively, resulting in a smaller detected voltage and a larger phase delay.^44,74–78^ Although Z1 remained the highest-signal sample in agar, Z2 retained a clearly detectable response after immobilization and showed stronger absolute harmonic signals than F1 and F2. This indicates that Z2 preserves useful MPI-relevant signal under constrained conditions, despite not being the strongest tracer in the freely dispersed state.

**Figure 7:**
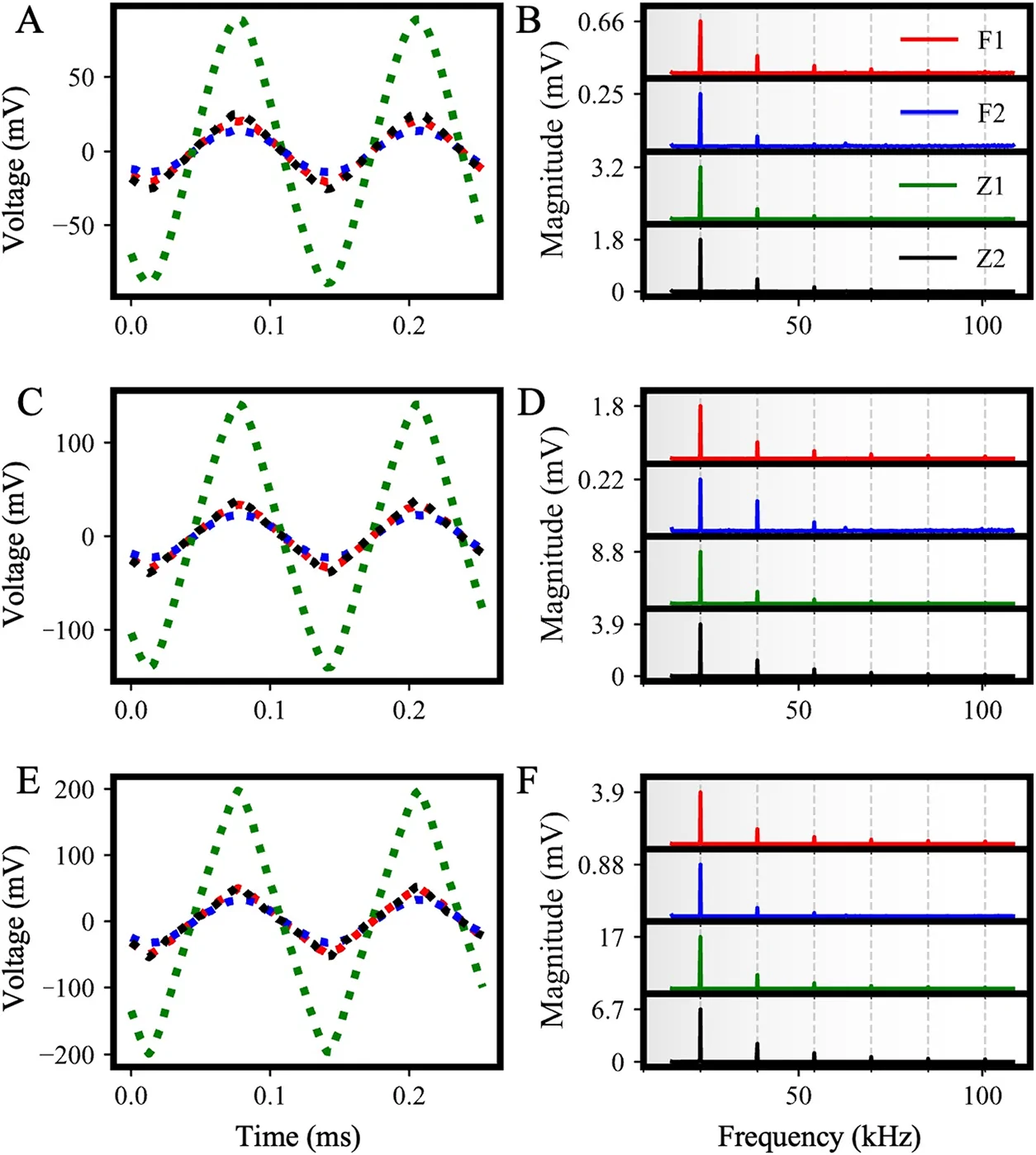
MPS characterization of MNP formulations embedded in the agar phantom. The excitation field is set at 7.75 kHz, and the amplitude is set at (A, B) 10 mT, (C, D) 15 mT, and (E, F) 20 mT, respectively. (A), (C), and (E) are the time-domain induced voltage signals collected from the receive coils. (B), (D), and (F) are the 3rd to 13th harmonic spectra; the amplitude of the 3rd component is labeled on the *y*-axis of each curve.

The harmonic spectra of the agar phantoms are shown in Figure 7B,D,F. Compared with the corresponding water phantoms’ spectra in Figure 6B,D,F, the agar phantoms’ spectra show lower harmonic amplitudes. This decrease indicates that immobilization reduces the nonlinear dynamic response needed for strong MPI signal generation. These results are informative for MPI cell tracking and tumor imaging, where particles may become immobilized after cellular uptake or tissue binding and therefore may not generate the same signal intensity observed in aqueous suspension.^24,61,79,80^ For Z2, the persistence of third, fifth, and seventh harmonic signals in agar suggests that the 10 nm Zn-substituted ferrite formulation can still provide MPI-detectable signal after Brownian motion is restricted, a feature that becomes important when considering Z2 as a dual-modal MPI-MRI tracer.

#### 3.5.3 Comparison of the Dynamic Magnetizations of MNPs in Water and Agar Phantoms

Figure 8 directly compares the MPI signal’s percentage changes between agar phantoms and water phantoms, using the water phantom response as the baseline. For all four MNP formulations, the harmonic amplitudes generally decreased after agar embedding. Figure 8A shows the percentage change in the third harmonic, Figure 8B shows the fifth harmonic, and Figure 8C shows the seventh harmonic. The percentage change in harmonic magnitude is defined as

1 $$\Delta A_{n}\;\left(\%\right)=\left({A_{n}|}_{agar}-{A_{n}|}_{water}\right)/{A_{n}|}_{water}\times 100\%$$

where *n* is the harmonic number.

**Figure 8:**
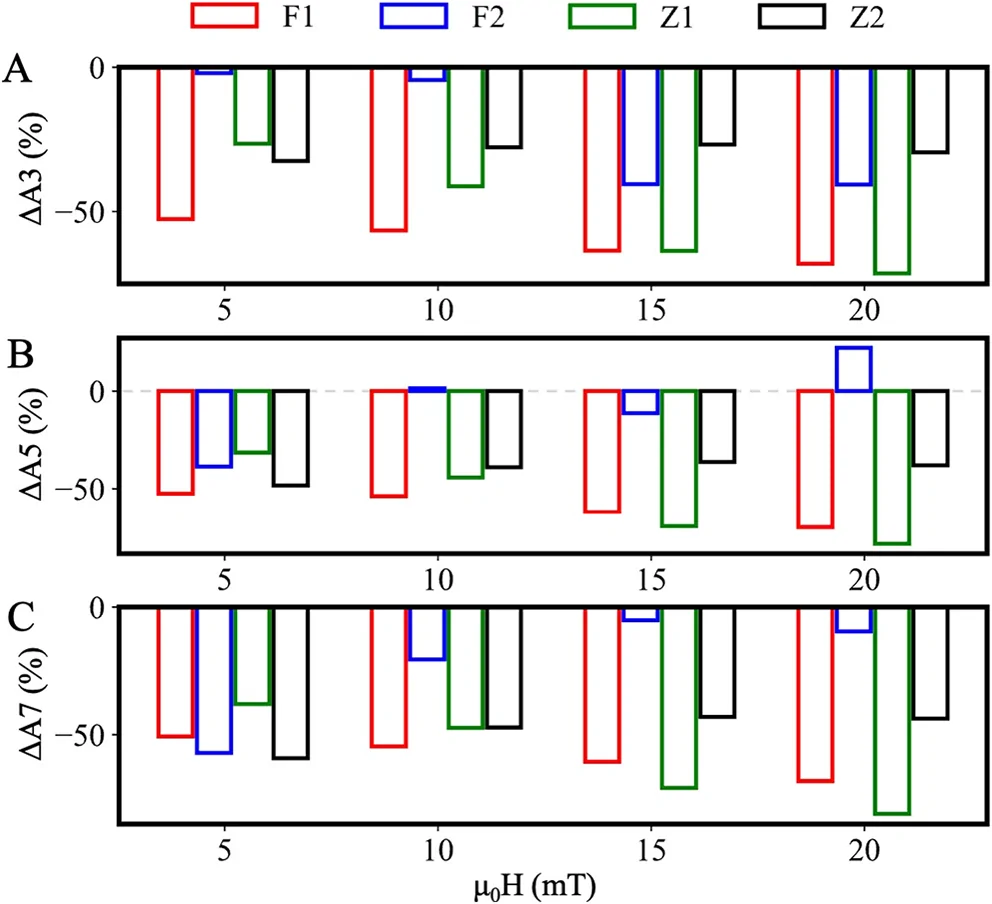
Relative changes in MPS harmonic amplitudes following agar embedding. Percentage change in (A) the 3rd, (B) the 5th, and (C) the 7th harmonic amplitudes. The excitation field is set at 7.75 kHz, and the amplitude is set at 5, 10, 15, and 20 mT.

Most samples showed harmonic signal losses ranging from 30% to 70%, depending on the particle formulation, harmonic order, and excitation field amplitude. F1 and Z1 generally showed larger decreases than the 10 nm formulations (F2 and Z2). This stronger sensitivity to agar immobilization is consistent with a larger Brownian contribution to the solution-state dynamic magnetic responses of the larger nanoparticles.^48,81,82^ When Brownian rotation is restricted by the agar matrix, their dynamic responses become more dependent on Néel relaxation, which may be less able to follow the excitation field at 7.75 kHz. This is physically reasonable because the Néel relaxation time increases exponentially with magnetic core volume, making larger particles more susceptible to signal loss when Brownian rotation is blocked.

The 10 nm MNPs behaved differently. F2 showed the smallest harmonic decrease overall and even displayed a slight increase in the fifth harmonic under selected conditions, as shown in Figure 8B. This behavior is consistent with a lower dependence on Brownian rotation. However, it does not indicate superior MPI-relevant performance because the absolute harmonic amplitudes of F2 were the lowest among the four formulations. Interestingly, Z2 showed a larger signal decrease than F2 in Figure 8. This difference indicates that Zn substitution changes the relaxation behavior of the 10 nm ferrite nanoparticles. However, the percentage decrease alone does not fully describe tracer performance as Z2 produced higher absolute harmonic amplitudes than F2 in both water and agar phantoms. Accordingly, absolute harmonic signal strength was considered the primary metric for evaluating MPI-relevant performance, whereas relative liquid-to-agar signal retention was considered a secondary metric reflecting the sensitivity of the tracer response to changes in the physical environment.

In summary, all four MNP formulations generated measurable higher harmonic signals and can therefore be considered MPI-relevant tracer candidates. However, their performance depends strongly on both nanoparticle structure and physical mobility. Z1 produced the strongest MPS response in the water phantom, as shown by the voltage traces and harmonic spectra, and remained the highest-signal sample in the agar phantom. Thus, Z1 exhibited the strongest absolute MPI-relevant signal under both freely dispersed and physically constrained conditions. Although F2 showed the smallest relative signal changes under most conditions, it produced the weakest absolute harmonic signals; thus, F2 is not suitable for MPI-relevant applications. Z2 occupies an intermediate position: it did not match Z1 in overall MPI signal strength, but it generated stronger absolute harmonic signals than F2 under both conditions while preserving detectable harmonic generation under agar embedding (Figure 8). Therefore, the water- and agar-phantom MPS measurements provide complementary information: the absolute harmonic amplitudes characterize MPI-relevant signal strength under each physical condition, whereas the relative changes provide secondary information about the environmental sensitivity of each MNP formulation. This distinction is important for dual-modal MPI-MRI tracer design since the optimal MPI-only tracer may differ from the optimal dual-modal tracer. When the T1-weighted MRI signal response is considered together with these MPS results, Z2 emerges as a promising balanced formulation because it combines good MPI-relevant harmonic generation with favorable, spatially localized MRI contrast behavior.

### 3.6 T1 Characterization of MNPs: Evaluation of MRI Performance

Here, T1-weighted MRI was performed using agar phantoms containing F1, F2, Z1, and Z2 at MNP concentrations of 100 and 500 μg/mL. As shown in Figure 9A, all four MNP formulations produced concentration-dependent signal attenuation compared to the water reference, producing hypointense signal under the tested T1-weighted imaging conditions. The signal became darker as the MNP concentration increased from 100 to 500 μg/mL, consistent with increased transverse relaxation and susceptibility-related signal loss at higher nanoparticle concentrations.^83,84^ Although the 30 nm MNP formulations (F1 and Z1) generated visible hypointense contrast, their signal darkening was accompanied by greater signal heterogeneity and more spatially extended hypointense regions. This effect was noticeable around the phantom inclusions, where the hypointense regions appeared to extend beyond the apparent boundaries of the samples. This behavior can be useful for qualitative detection, but it may complicate accurate boundary definition and quantitative signal intensity analysis.^85,86^ In contrast, the 10 nm MNP formulations (F2 and Z2) produced more localized and better-defined signal attenuation. Their smaller magnetic cores may generate less extensive long-range susceptibility perturbation, allowing water protons to experience a more spatially confined relaxation effect. This finding suggests that F2 and Z2 may be more favorable under T1-weighted imaging conditions where spatial localization and boundary definition are important.

**Figure 9:**
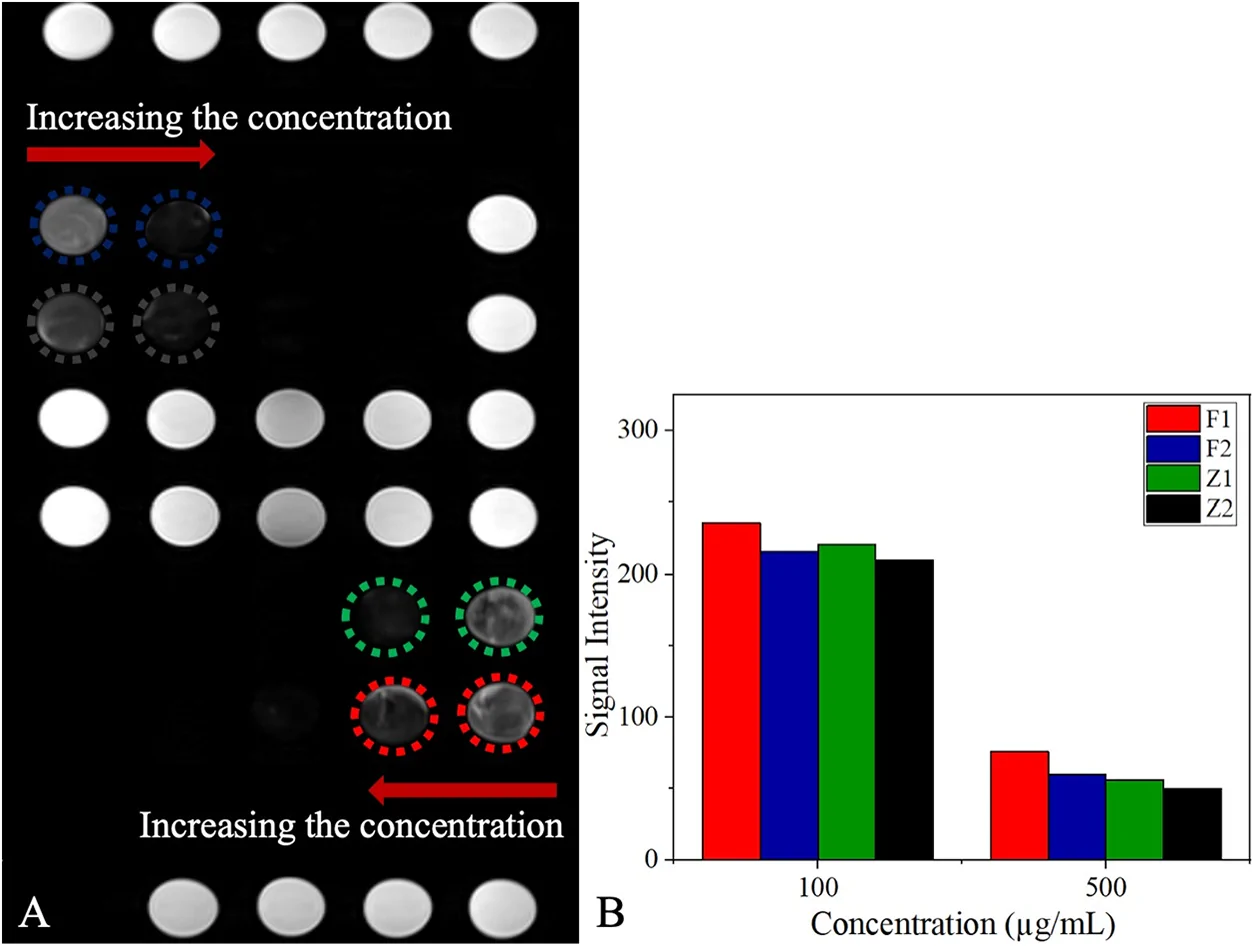
(A) T1-weighted MRI images of agar phantoms containing Z1 (green dashed circles), Z2 (black-gray dashed circles), F1 (red dashed circles), and F2 (blue dashed circles) at iron concentrations of 100 and 500 μg/mL. (B) Mean T1-weighted MRI signal intensities of four MNP-containing agar phantoms as a function of MNP concentration.

The quantitative signal analysis in Figure 9B further confirms the concentration-dependent MRI response of all four formulations. As the MNP concentration increased from 100 to 500 μg/mL, the signal intensity decreased for each MNP formulation, with Z2 showing the strongest overall signal attenuation under T1-weighted imaging conditions among the four samples. F2 also showed substantial signal reduction, but its attenuation was weaker than that of Z2, indicating that Zn substitution improves the MRI contrast performance of the 10 nm ferrite nanoparticles. Therefore, among the four formulations, Z2 provides the most favorable MRI performance by combining strong pronounced signal attenuation with a more spatially confined contrast pattern.

### 3.7 Considerations for Dual-Modal MPI-MRI Tracers

Table 1 summarizes the representative MPI- and MRI-relevant signal metrics of the four MNP formulations, including the third, fifth, and seventh harmonic amplitudes measured by MPS in water and agar phantoms. The harmonic amplitudes were normalized to the MNP mass and are reported in mV/mg MNP, together with the corresponding averaged T1-weighted MRI signal intensities. The table shows distinct formulation-dependent performance patterns across the MPI and MRI measurements. In solution (water phantom), Z1 generated the strongest mass-normalized harmonic signals, confirming it as the highest-signal MPI tracer under freely dispersed conditions. After agar embedding (agar phantom), Z1 is still the strongest formulation across all three harmonics, although its harmonic amplitudes decreased substantially, consistent with sensitivity to matrix-induced physical restriction. In comparison, Z2 produced lower absolute signals than Z1 but the second-highest mass-normalized harmonic amplitudes in the agar phantom, outperforming both F1 and F2 across all three harmonics. Accordingly, the absolute harmonic signal strength was considered the primary metric for evaluating MPI-relevant performance. Relative liquid-to-agar signal retention was considered a secondary metric reflecting the robustness of the tracer response to changes in the physical environment, including conditions in which the MNPs become immobilized or physically constrained.

**Table 1:**
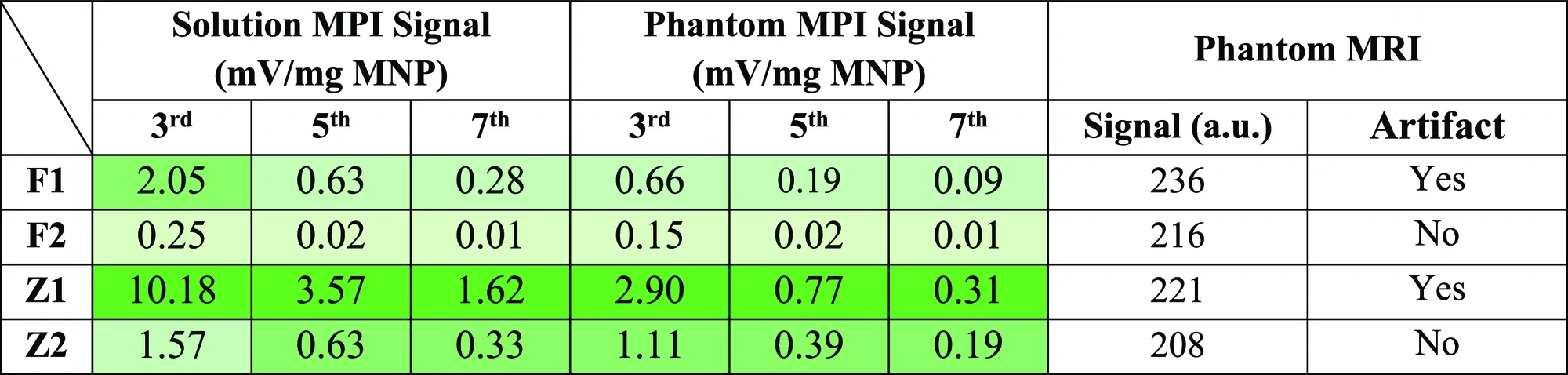
Summary of MPS-Derived MPI-Relevant Harmonic Signals and T1-Weighted MRI Responses of the Four MNP Formulations^a^

a The harmonic amplitudes were normalized to the MNP mass and are reported in mV/mg MNP. In the MPS harmonic-amplitude columns, darker (lighter) green indicates a stronger (weaker) mass-normalized signal. For quantitative T1-weighted MRI signal-intensity analysis, each circular sample region was divided into five regions of interest (ROIs), and the signal intensity of each ROI was measured and averaged for each sample; lower values indicated greater hypointense signal attenuation. Artifact refers to hypointense regions consistent with susceptibility-related artifacts.

Table 1 also supports the dual-modal interpretation of MPI-MRI performance. Although Z1 exhibited the highest mass-normalized MPS harmonic amplitudes in both solution and agar phantoms, its MRI contrast was accompanied by spatially extended hypointensity, which can reduce boundary definition. In contrast, Z2 combined the lowest average T1-weighted MRI signal intensity with a more spatially localized hypointense contrast pattern and the second-highest agar-phantom harmonic amplitudes. This comparison defines a clear design trade-off: the 30 nm Zn-substituted sample Z1 favors maximum MPI-relevant signal strength, whereas the 10 nm Zn-substituted sample Z2 provides a better balance between MPI detectability and spatially localized MRI contrast. Therefore, Z2 emerges as the most promising balanced candidate for dual-modal MPI-MRI imaging among the four formulations, whereas Z1 remains the strongest MPI-relevant formulation under both freely dispersed and agar-embedded conditions.

## 4 Conclusion

In this work, we synthesized and systematically evaluated four citrate-functionalized, quasi-cubic ferrite nanoparticle formulations: 30 nm Fe_3_O_4_ (F1), 10 nm Fe_3_O_4_ (F2), 30 nm Zn_0.4_Fe_2.6_O_4_ (Z1), and 10 nm Zn_0.4_Fe_2.6_O_4_ (Z2), as candidate tracers for dual-modal MPI-MRI imaging. TEM, XRD, DLS, FTIR, and EDX confirmed that the particles possessed the intended size, morphology, spinel ferrite structure, citrate-associated surface chemistry, and Zn incorporation in the doped formulations. Static magnetization measurements showed that both core size and Zn substitution modulated the magnetic response, with the larger particles and Zn-substituted ferrites exhibiting stronger magnetization while maintaining relatively low coercivity.

MPS measurements further revealed that MPI-relevant performance depends not only on composition and size, but also on the physical state of the nanoparticles. Z1 produced the highest mass-normalized third, fifth, and seventh-harmonic amplitudes in both water and agar phantoms, identifying it as the highest MPI signal formulation under both freely dispersed and agar-embedded conditions. Agar embedding reduced the harmonic amplitudes, consistent with restriction of Brownian rotation and alteration of the dynamic magnetization response. Z2 produced the second-highest MPI signals in the agar phantom and outperformed F2 among the 10 nm formulations, demonstrating the benefit of Zn substitution under physically constrained conditions. The relative solution-to-agar signal changes of MNPs were considered as secondary indicators of sensitivity to immobilization in this work to assist the evaluation of MPI performance. Our results identify Z1 as the strongest MPI signal formulation and that tracers should be evaluated directly under the physical conditions relevant to their intended biological application.

The biological and MRI results further support the potential of these MNPs for dual-modal imaging. Live/Dead assays demonstrated minimal acute cytotoxicity in both SKOV3 and MCF7 cells after 24 h incubation, and bright-field imaging confirmed concentration-dependent nanoparticle association with SKOV3 cells. T1-weighted MRI phantom imaging showed that all four MNP formulations exhibited hypointense signal attenuation. The 10 nm formulations (Z2 and F2) generated more spatially localized hypointense contrast, whereas the 30 nm formulations (Z1 and F1) generated susceptibility-induced signal loss extending beyond the physical inclusion. This spatially extended hypointensity made the larger formulations less favorable for boundary delineation and quantitative signal-intensity analysis under the tested T1-weighted imaging conditions.

In conclusion, our study establishes a structure-magnetic property-imaging performance relationship for Fe_3_O_4_ and Zn-substituted ferrite nanoparticles. Z1 provides the strongest MPI-relevant signal when maximizing absolute harmonic amplitude is the primary objective, whereas Z2 offers a more favorable balance between MPI-relevant signal strength and spatially localized T1-weighted MRI contrast. Among the four MNP formulations investigated, Z2 therefore represents the most promising balanced candidate for dual-modal MPI-MRI imaging, while Z1 remains the strongest candidate for applications prioritizing MPI signal strength.
